# Systematic review and meta‐analysis comparing outcomes following orchidopexy for cryptorchidism before or after 1 year of age

**DOI:** 10.1002/bjs5.36

**Published:** 2018-02-05

**Authors:** B. S. R. Allin, E. Dumann, D. Fawkner‐Corbett, C. Kwok, C. Skerritt, on behalf of the Paediatric Surgery Trainees Research Network

**Affiliations:** ^1^ National Perinatal Epidemiology Unit University of Oxford Oxford UK; ^2^ Medical Sciences Division University of Oxford Oxford UK; ^3^ Oxford Children's Hospital Oxford UK; ^4^ Evelina Children's Hospital London UK

## Abstract

**Background:**

Current guidelines recommend orchidopexy for cryptorchidism by 12 months of age, yet this is not universally adhered to. The aim of this systematic review and meta‐analysis was to compare outcomes between orchidopexies performed before and after 1 year of age.

**Methods:**

MEDLINE and Embase were searched (September 2015) using terms relating to cryptorchidism, orchidopexy and the outcomes of interest. Studies were eligible for inclusion if they compared orchidopexy at less than 1 year of age (early) with orchidopexy at 1 year or more of age (delayed) and reported the primary outcome (testicular atrophy) or one of the secondary outcomes (fertility potential, postoperative complication, malignancy). Studies were excluded when more than 50 per cent of infants had intra‐abdominal testes, or the population included infants with disorders of sexual differentiation. Additional studies were identified through reference list searching. Unpublished data were sought from the ORCHESTRA study investigators.

**Results:**

Fifteen eligible studies were identified from 1387 titles. There was no difference in atrophy rate between early orchidopexy and delayed orchidopexy (risk ratio 0·64, 95 per cent c.i. 0·25 to 1·66; 912 testes). Testicular volume was greater (mean difference 0·06 (95 per cent c.i. 0·01 to 0·10) ml; 346 testes) and there were more spermatogonia per tubule (mean difference 0·47 (0·31 to 0·64); 382 testes) in infants undergoing early orchidopexy, with no difference in complication rate (risk ratio 0·68, 0·27 to 1·68; 426 testes). No study reported malignancy rate.

**Conclusion:**

Atrophy and complication rates do not appear different between early and delayed orchidopexy, and fertility potential may be better with early orchidopexy. Imprecision of the available data limits the robustness of these conclusions.

## Introduction

Cryptorchidism affects between 2·4 and 5 per cent of live‐born boys^1^, ^2^, ^3^, ^4^. Undescended testes are known to be associated with an increased incidence of testicular malignancy and subfertility, even after orchidopexy^5^, ^6^, ^7^, ^8^, ^9^, ^10^. Epidemiological evidence^11^ shows that the risk of testicular cancer is increased markedly when orchidopexy is delayed to 13 years of age. However, histological changes are evident in testicular biopsies from boys as young as 2 years^10^.

The current theory is that there is a short time frame between 3 and 6 months of age when the normal maturation of gonocytes into type A spermatogonia should occur, and this process is temperature sensitive^12^. During this process there is also apoptosis of germ cells, and it is postulated that these persistent undifferentiated germ cells may lead to malignancy in the long term^13^. The latest guidance from several professional associations^14^, ^15^, ^16^, ^17^, including a recent British Association of Paediatric Urologists (BAPU) consensus statement, recommends performing orchidopexy by 12 months of age.

This guidance is not adhered to universally. A recent international survey^18^ of 122 paediatric surgeons and urologists found that around half of surgeons considered that the optimal age for orchidopexy should be slightly older. Reasons cited for delaying surgery until boys are older than 12 months were concerns about possible increased rates of postoperative testicular atrophy in younger children and the heightened awareness of possible adverse effects on neurodevelopment of general anaesthesia in very young children. Much of the evidence about effects of anaesthesia on the developing brain come from animal studies; however, there is an increasing body of research investigating possible effects in humans^19^, ^20^, ^21^, ^22^.

A recent systematic review by Chan and colleagues^23^ found some evidence to support earlier orchidopexy. However, this review was limited in terms of utility for supporting the BAPU consensus statement, as it did not specifically examine the merits of using a 1‐year age cut‐off, and provided limited evidence in relation to the harms that may be associated with earlier orchidopexy. The aim of the present systematic review was therefore to build upon the work of Chan *et al*.^23^ by making a specific comparison of the outcomes associated with orchidopexy before or after 1 year of age, including both benefits and harms.

## Methods

The review was conducted according to a prespecified protocol registered on the Prospero International Prospective Register of Systematic Reviews (CRD42016025930).

The population of interest for this review were boys without disorders of sexual differentiation who were identified as having unilateral or bilateral cryptorchidism, and for whom an orchidopexy was performed. The intervention of interest was orchidopexy performed before 1 year of age, with orchidopexy performed at 1 year of age or later as a comparator. The primary outcome was testicular atrophy, regardless of method of measurement, with secondary outcomes including fertility potential, testicular malignancy, neurodevelopmental outcomes, and surgical or anaesthetic complication (defined as any deviation from the usual, anticipated, postoperative course). Studies were therefore eligible for inclusion if they compared orchidopexy performed before 1 year of age with orchidopexy performed at or after 1 year of age in boys with either unilateral or bilateral cryptorchidism, and reported at least one outcome of interest. It was considered likely that the outcomes of interest would be reported using multiple different methods of measurement, at multiple different time points. As the intention of this review was to understand the current state of evidence, as opposed to drawing robust conclusions for management, the decision was made not to restrict eligibility of studies based on their use of a specific outcome measure. Meta‐analysis was, however, performed only for studies reporting comparable outcome measures.

As it is believed that infants with intra‐abdominal testes have worse outcomes than those with testes located in the inguinal canal or suprascrotal pouch, studies that assessed outcomes purely for infants undergoing treatment for intra‐abdominal cryptorchidism were excluded from the review. Studies where there was a mixed population of infants were, however, included, if the majority of testes were in the inguinal canal or suprascrotal pouch. This decision was made, as it was considered that exclusion of all studies with a heterogeneous population would prevent the review from addressing its primary objective. To aid transparency of the comparability of the intervention and control groups, the authors sought to describe the testicular positions reported in each included study. Studies including infants with disorders of sexual differentiation were also excluded, as these children are likely to represent a different population to those without disorders of sexual differentiation.

Multiple search strategies were used to identify relevant articles from MEDLINE and Embase. The initial search was performed on 3 September 2015, and rerun on 15 December 2016 to confirm that no additional eligible articles had been published between the initial search and submission for publication. Search terms were identified from database thesauri and free text relating to cryptorchidism, orchidopexy and the outcomes of interest. Terms were combined using Boolean operators (*Appendix S1*, supporting information). All study designs, except expert opinion, were eligible for inclusion, and no limits were placed based on year of publication, language or geographical location. Hand‐searching of reference lists from included manuscripts was undertaken to identify additional eligible studies.

All papers were reviewed for eligibility by two authors working independently. Discrepancies were resolved by discussion, with recourse to another author when necessary. Data were also extracted by two authors working independently, again with discrepancies resolved by discussion and recourse to another author if needed.

Additional data were sought from the ORCHESTRA study group. The ORCHESTRA study^24^ is a multicentre, international cohort study comparing atrophy rates in infants undergoing orchidopexy before 1 year of age with infants undergoing orchidopexy at or after the age of 1 year. This study is known to have concluded data collection, but has not yet published its results.

As recommended by the Cochrane Collaboration, the quality of the body of evidence contributing to each outcome was assessed by means of Grading of Recommendations Assessment, Development and Evaluation (GRADE). A summary for each outcome, which could range from very low to high quality, was produced.

### Statistical analysis

Mean differences were calculated for continuous variables using the inverse variance method. Risk ratios (RRs) were calculated for dichotomous variables using the Mantel–Haenszel method. Meta‐analyses were performed using Review Manager (RevMan) version 5.3 (The Cochrane Collaboration, The Nordic Cochrane Centre, Copenhagen, Denmark).

Where studies reported continuous outcomes using mean(s.d.) value for subgroups of the intervention and control groups used in the present review, data from these subgroups were combined into one summary measure using Microsoft® Excel® for Mac™ 2011 version 14.7.4 (170508) (Microsoft, Redmond, Washington, USA).

## Results

### Included studies and quality of evidence

**Figure 1:**
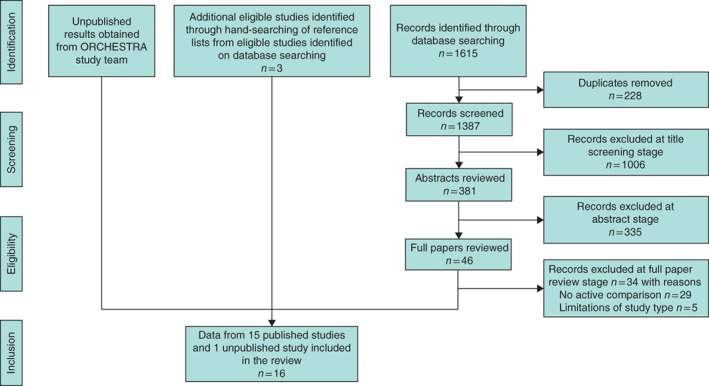
PRISMA diagram for the study

After removal of duplicates, 1387 titles were reviewed, with 1006 excluded at title review stage, 335 at abstract review and 34 on review of full papers (*Table S1*, supporting information). After hand‐searching of the reference lists of the 12 eligible studies identified from database searching, a further three eligible studies were identified. In total, 15 published studies^9^ ^25^, ^26^, ^27^, ^28^, ^29^, ^30^, ^31^, ^32^, ^33^, ^34^, ^35^, ^36^, ^37^, ^38^ were included in the review (*Fig*. 1 and *Table* 1). Of the 15 papers, four by Kollin and co‐workers^25^, ^26^, ^27^, ^28^ were based on the same population of infants, and papers by Feyles *et al*.^29^ and Canavese and colleagues^9^ ^30^, ^31^ also had overlapping populations. There was one RCT reported in four studies^25^, ^26^, ^27^, ^28^ at different time points, three prospective cohort studies^30^ ^32^, ^33^, one case–control study^34^, one retrospective cohort study nested within a larger case–control study^35^ and six retrospective cohort studies or case series^9^ ^29^, ^31^ ^36^, ^37^, ^38^. A retrospective cohort study by Tasian *et al*.^37^ was also reported as a conference abstract^39^ containing data from 14 more participants than the full manuscript. As the abstract contained significantly less detail than the full manuscript, and was reporting the same population, it was not included in the review. Unpublished data from the ORCHESTRA study^24^ were included in the analysis of the primary outcome.

**Table 1:** Characteristics of included studies

		Age at orchidopexy		No. of testes*			
Reference	Setting and methodology	Intervention	Comparator	Intervention	Control	No. of intra‐abdominal testes†	Outcomes reported
Kogan et al.^32^	Prospective cohort	< 12 months	12–46 months	13	64	0 (0)	Postoperative complication, testicular atrophy, testicular retraction, anaesthetic complication, mean seminiferous tubule diameter, mean number of germ cells per tubule
Canavese et al.^31^	Retrospective cohort study	< 12 months	> 12 months	84	832	65 (7·1) in entire cohort	Testicular morphology, percentage of testes with normal number of spermatogonia
	Single centre						
	Recruited 1980–1992						
McAleer et al.^38^	Retrospective cohort study	< 12 months	1–16 years	51‡	189‡	25 (9·3) in entire cohort	Fertility index (mean number of spermatogonia per tubule)
	Single centre						
	Recruited 1986–1990						
Lala et al.^33^	Prospective cohort study Single centre Unclear overlap of population with Canavese study^9^ ^30^, ^31^	< 12 months, with or without failed LH and HCG therapy	> 12 months, after failed LH and HCG therapy	52	155	Intervention group 14 (27) Control group 32 (20·6)	Descent rate with hormone therapy, hormone therapy side‐effects, duration of surgery, anaesthetic complications, postoperative complications, tubular atrophy, Leydig cell atrophy, normal epithelial histology, tubular atrophy, number of spermatogonia and type Ad spermatogonia
							
							
Canavese et al.^30^	Prospective cohort study Single centre Recruitment period not specified	< 12 months	12–36 months	82 (67)	72 (60)	Unable to determine from graphical representation	Testicular morphology, number of spermatogonia and type Ad spermatogonia, tubular atrophy, Leydig cell atrophy, and testicular atrophy
							
							
Kollin et al.^25^	RCT with significant methodological limitations	9 months	3 years	66 (70)§	69 (79)§	0 (0)	Median volume increase in testicular size between birth and age 2 years, ratio of descended to undescended testis volume, testicular atrophy, reoperation
Kollin et al.^26^	Follow‐up of Kollin et al.^24^; additional patients recruited	9 months	3 years	67 (72)§	72 (83)§	0 (0)	Testicular growth from birth to age 4 years, ratio of descended to undescended testis volume
Park et al.^34^	Case–control studySingle centreRecruitment 1998–2001	< 12 months	> 12 months	20 (20)	45 (45)	n.r.	Number of germ cells per tubule, interstitial peritubular fibrosis, mean tubular fertility index, germ cell count, testicular volume at surgery, mean tubular diameter, Sertoli cell index
							
							
Canavese et al.^9^	Retrospective cohort studySingle surgical centreRecruitment 1986–1991	< 12 months	12–24 months	18 (13)	18 (16)	Intervention group 1 (6)Control group 3 (17)	Testicular volume at surgery, testicular volume at follow‐up, sperm count > 20 million/ml, normal total sperm count, highly motile spermatozoa, sperm motility
							
							
Tasian et al.^37^	Retrospective cohort studySingle centreRecruitment 1991–2001	< 12 months	12 months to 18 years	274 patients in entire cohort		45 (16·4) in entire cohort	Odds of germ cell depletion per month of age at operation, odds of Leydig cell absence, severity of fibrosis
							
							
Kollin et al.^27^	Follow‐up of Kollin et al.^25^; additional patients recruited and 8 non‐randomized patients included in intervention group	9 months	3 years	127¶	92¶	Intervention group 22 (17·3)Control group 10 (11)	Mean testicular volume at surgery, Sertoli cells per 100 cords, germ cells per 100 cords, cord diameter, percentage interstitial tissue, serum FSH, LH, inhibin B and testosterone levels
							
Kollin et al.^28^	Follow‐up of Kollin et al.^25^	9 months	3 years	(78)#	(85)#	n.r. (assumed the same as Kollin et al.^25^)	Testicular volume at follow‐up
Van Brakel et al.^35^	Retrospective cohort study nested within a case–control study of impact of cryptorchidism on markers of fertility	< 12 months	12 months to 12 years	(8)**	(36)**	n.r.	Testicular volume at follow‐up, serum LH, FSH, testosterone and inhibin B levels, sperm concentration
Carson et al.^36^	Retrospective cohort study	< 12 months	1–16 years	64	285	50 (14·3) in entire cohort	Testicular atrophy, postoperative complications
	Single centre						
	Recruitment 2000–2010						
Feyles et al.^29^	Retrospective cohort studySingle centreRecruitment 1986–1993Population overlaps with Canavese study^9^ ^30^, ^31^	< 12 months	12–24 months	35 (27)	27 (24)	Intervention group 6 (17)	Testicular volume at surgery, testicular volume at follow‐up, total sperm count > 15 million/ml, highly motile spermatozoa, normal sperm count (%), normal sperm motility (%)
							
							
						Control group 2 (7)	
ORCHESTRA study^24^	Prospective cohort study	< 12 months	≥ 12 months	39 (39)	303 (303)	0 (0)	Postoperative testicular atrophy
	Multicentre						
	Recruitment 3‐month period in 2014						

Values in parentheses are

* number of infants and

† percentage of total testes unless indicated otherwise.

‡ A total of 268 testes were recruited, but only 240 were analysed in primary study owing to inadequacy of samples;

§ number of infants randomized, with number of testes available at first follow‐up time point in parentheses;

¶ number of testes per group clear, but number of infants in each group unclear;

# exact numbers of patients and testes unclear as text differs from tables;

** 62 boys with cryptorchidism included and 53 healthy controls recruited, but number of infants in intervention and control groups not stated.

Of the included studies, four^24^, ^25^, ^26^ ^32^ did not include any infants with intra‐abdominal testes, eight^9^ ^27^,^29^ ^31^, ^33^ ^36^, ^37^, ^38^ had less than 50 per cent intra‐abdominal testes in their cohort, and four studies^28^ ^30^, ^34^ ^35^ did not report the proportion of intra‐abdominal testes.

The GRADE quality of evidence was assessed as very low for all reported outcomes. All bar one of the studies contributing to the outcomes were observational in nature, and therefore the maximum score that could be awarded was low‐quality evidence. Each study was judged to have sufficient methodological limitations to warrant downgrading to very low‐quality evidence. Limitations included unclear comparability of baseline populations between intervention and control groups, imprecision of study results due to their small size, limitations associated with retrospective study designs, and unclear influence of loss to follow‐up. The one RCT^25^ included in the review was judged as very low‐quality evidence owing to a lack of allocation concealment, lack of blinding, unclear loss to follow‐up and inclusion of non‐randomized participants in the study.

### Testicular atrophy

**Figure 2:**
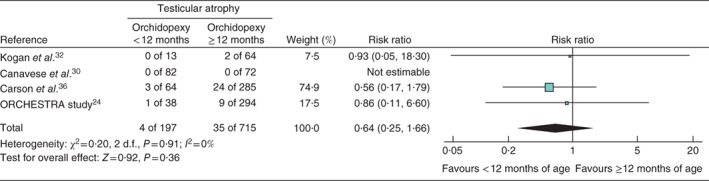
Forest plot comparing testicular atrophy in boys with cryptorchidism who underwent orchidopexy at less than 1 year of age with those who had the operation at or after the age of 1 year. A Mantel–Haenszel fixed‐effect model was used. Risk ratios are shown with 95 per cent confidence intervals

Five studies^24^ ^25^, ^30^ ^32^, ^36^ reported postoperative atrophy rates, with different definitions of atrophy used. Kollin and colleagues^25^ defined atrophy according to a reduction in ultrasound‐measured testicular volume, Carson and co‐workers^36^ and the ORCHESTRA study^24^ defined atrophy as greater than 50 per cent reduction in postoperative size compared with either preoperative examination or unaffected contralateral testis, and Canavese et al. 30 and Kogan et al. 32 did not define atrophy at all. Only four of these studies^24^ ^30^, ^32^ ^36^ were included in the meta‐analysis, as Kollin and co‐workers^25^ reported atrophy rates only for the intervention group. In total, 912 testes were included in the meta‐analysis; 197 orchidopexies were performed before 12 months of age, with four reported cases of atrophy (2·0 per cent), and 715 orchidopexies were performed after 1 year of age, with 35 cases of atrophy reported (4·9 per cent) (RR 0·64, 95 per cent c.i. 0·25 to 1·66) (Fig. 2).

### Postoperative complications

**Figure 3:**
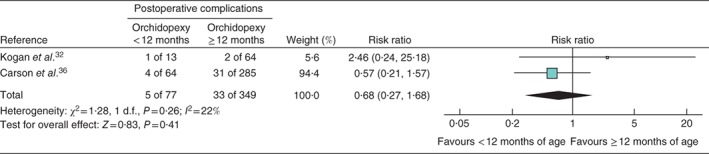
Forest plot comparing grouped postoperative complications, including testicular atrophy, in boys with cryptorchidism who underwent orchidopexy at less than 1 year of age with those who had the operation at or after the age of 1 year. A Mantel–Haenszel fixed‐effect model was used. Risk ratios are shown with 95 per cent confidence intervals

Three papers^32^ ^33^, ^36^ reported rates of postoperative complications as a grouped measure including testicular atrophy. However, as Lala and colleagues^33^ reported complications only for the intervention group, data from this study were not included in the meta‐analysis. Overall, of 77 orchidopexies performed before 1 year of age, five complications occurred (6 per cent), whereas 33 complications occurred following the 349 orchidopexies performed after 1 year of age (9·5 per cent) (RR 0·68, 95 per cent c.i. 0·27 to 1·68) (*Fig*. 3).

In 13 infants undergoing orchidopexy before 1 year of age, Kogan et al. 32 reported one wound infection (8 per cent), and in the 64 infants undergoing orchidopexy at or after 1 year of age they reported one episode of testicular ascent requiring repeat orchidopexy (2 per cent) and one episode of testicular atrophy (2 per cent). Carson and colleagues^36^ described the complications only for the whole cohort: for a total of 349 orchidopexies, they reported 27 episodes of testicular atrophy (7·7 per cent), nine episodes of testicular ascent (2·6 per cent), four wound infections (1·1 per cent) and one hernia (0·3 per cent), giving a total of 41 complications reported in 35 children.

### Spermatogonia, tubular diameter and testicular volume at surgery

**Figure 4:**
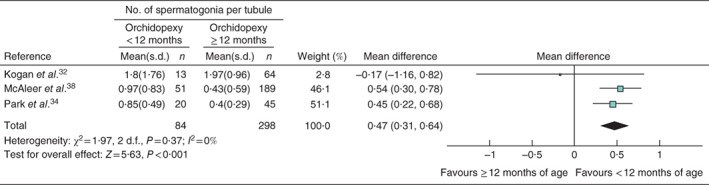
Forest plot comparing the number of spermatogonia per tubule in boys with cryptorchidism who underwent orchidopexy at less than 1 year of age with those who had the operation at or after the age of 1 year. An inverse‐variance fixed‐effect model was used. Mean differences are shown with 95 per cent confidence intervals

Studies reported multiple surrogate measures of fertility. The most common were mean number of spermatogonia per seminiferous tubule, mean tubular diameter and testicular volume at the time of surgery. Three studies^32^ ^34^, ^38^ reported the mean number of spermatogonia per tubule, contributing 84 testes to the intervention group and 298 to the control group. There were more spermatogonia per tubule in infants undergoing orchidopexy before 12 months of age than in those having orchidopexy at 12 months of age or later (mean difference 0·47, 95 per cent c.i. 0·31 to 0·64) (Fig. 4).

**Figure 5:**
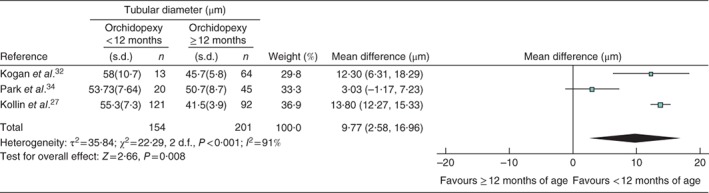
Forest plot comparing tubular diameter in boys with cryptorchidism who underwent orchidopexy at less than 1 year of age with those who had the operation at or after the age of 1 year. An inverse‐variance random‐effects model was used. Mean differences are shown with 95 per cent confidence intervals

Three studies^27^ ^32^, ^34^ reported mean tubular diameter at time of study, contributing 154 testes to the intervention group and 201 to the control group. Tubular diameter in infants undergoing orchidopexy before 12 months of age was larger than that in the older children by a mean difference of 9·77 (95 per cent c.i. 2·58 to 16·96) μm (*Fig*. 5).

**Figure 6:**
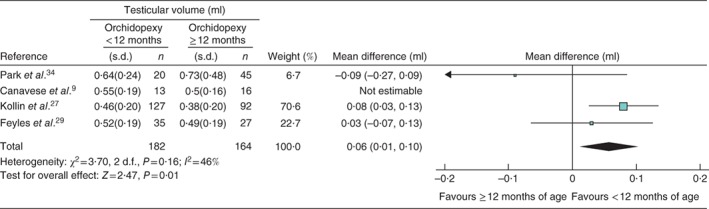
Forest plot comparing testicular volume at surgery in boys with cryptorchidism who underwent orchidopexy at less than 1 year of age with those who had the operation at or after the age of 1 year. An inverse‐variance fixed‐effect model was used. Mean differences are shown with 95 per cent confidence intervals

Four studies^9^ ^27^, ^29^ ^34^ reported testicular volume at the time of surgery; however, two^9^ ^29^ reported data from the same population of infants, and therefore data from the smaller of these was not used in the meta‐analysis. Testicular volume at the time of surgery was reported for 182 testes in the intervention group and 164 in the control group. The mean difference in volume at the time of surgery was 0·06 (95 per cent c.i. 0·01 to 0·10) ml greater for the intervention group (Fig. 6).

### Testicular volume at follow‐up

Testicular volume at follow‐up was reported in five studies^9^ ^25^, ^26^ ^28^, ^29^. Kollin and colleagues conducted an RCT in which infants were randomized at 6 months of age to orchidopexy at either age 9 months or 3 years. In a series of three manuscripts^25^ ^26^, ^28^, published in 2006, 2007 and 2013, they reported testicular size in both the intervention and control groups at different ages. In the 2006 report^25^, boys in the delayed surgery group were still preoperative. In the 2007 report^26^, both the early and delayed surgery groups were postoperative, and it was found that orchidopexy at 9 months of age resulted in a statistically significantly larger testis at age 4  years than when orchidopexy was performed at age 3 years. The data, however, are reported as median (i.q.r.) values, and therefore cannot be subjected to meta‐analysis.

In their final 2013 report, Kollin *et al*.^28^ followed the same cohort of children up to age 5 years; boys who had undergone orchidopexy at 9 months of age had a statistically significantly larger testis at age 5 years than those who had orchidopexy at the age of 3 years. Canavese and co‐workers^9^ and Feyles *et al*.^29^ did not find any difference in mean testicular volume between the early and delayed surgery groups at follow‐up. These studies, however, were both significantly smaller than that of Kollin *et al*., and followed the children through into adulthood rather than stopping follow‐up at age 5 years. Both of these studies^9^ ^29^ drew data from the same population of children, and the Feyles study^29^ was the larger of the two. Given that these two papers reported on the same population, and their average age at follow‐up was different from that of the Kollin cohort^28^ by approximately 15 years, meta‐analysis was not performed. It was not possible to meta‐analyse data reported in either of the other studies^25^ ^26^, so no summary measure has been produced for testicular volume at follow‐up.

### Other fertility outcomes

Both Canavese and colleagues^31^ in 1993 and Feyles and co‐workers^29^ in 2014 reported that boys undergoing orchidopexy before 1 year of age were more likely to have a normal total sperm count in later life than those having orchidopexy at or after age 1 year. In 2009, Canavese *et al*.^9^ reported that infants undergoing orchidopexy before the age of 1 year had a higher total sperm count, which was more likely to be greater than 20 million per ml in later life than boys undergoing orchidopexy at or after 1 year of age. All three studies^9^ ^29^, ^31^, however, drew data from the same population of infants; therefore, the results are not independent and meta‐analysis was inappropriate.

Other fertility‐related outcome measures reported in eligible studies include the number of spermatogonia per tubule (fertility index)^38^, percentage of tubules containing at least one germ cell amongst 50 randomly selected tubules, mean number of Sertoli cells per 50 randomly selected tubules, mean tubular diameter^34^, number of Sertoli cells per 100 tubules, number of germ cells per 100 tubules^27^, tubular atrophy^30^ and sperm motility^29^. Although many of these measures are reported to be better in infants undergoing orchidopexy before 1 year of age, there was such heterogeneity that meta‐analysis to summarize them was not possible.

### Other outcomes

There were no studies reporting outcomes directly relating to malignancy or neurodevelopment.

## Discussion

This systematic review and meta‐analysis comparing outcomes following orchidopexy before 1 year of age with outcomes following orchidopexy at or after the age of 1 year for cryptorchidism included 15 published studies and data from one unpublished study. Seven of the published studies were drawn from the same two populations of infants, and the quality of evidence provided for all reported outcomes was graded as very low. Four studies^24,30,32,36^ including 912 testes contributed data to analysis of the primary outcome, testicular atrophy, with no difference in rates seen between boys who underwent orchidopexy before 1 year of age and those who had orchidopexy at or after 1 year of age.

There was also no evidence of difference in overall rates of postoperative complications between the two groups. Meta‐analysis of secondary outcomes relating to fertility suggested that boys who underwent orchidopexy before 1 year of age had a statistically significantly larger testicle at the time of orchidopexy, significantly larger‐diameter seminiferous tubules, and a significantly higher number of spermatogonia per seminiferous tubule than those who had orchidopexy at or after 1 year of age. Although not suitable for meta‐analysis, other markers of fertility potential reported in eligible studies also appeared to suggest greater fertility potential in infants who underwent orchidopexy before 1 year of age.

These results therefore suggest a potential benefit of performing orchidopexy before 1 year of age, with no current evidence of additional harm. These results must, however, take into consideration the robustness of the primary data included in the review.

Although robust methodology was used in the design of this study, there are multiple limitations that affect interpretation of the findings. First, no limits were placed on the age at which orchidopexy at or after 1 year of age could be performed, making the control group particularly heterogeneous. Further heterogeneity was introduced as boys with intra‐abdominal cryptorchidism and those who had undergone hormonal therapy before orchidopexy were included in the review. Distribution of these children amongst the control and intervention groups was not always clearly described, making it difficult to interpret how these factors may have confounded the review's results. Patient flow and study methodology were also unclear in many of the included studies, making it difficult to assess the impact of loss to follow‐up and potential chance, confounding and bias on the results of the review.

Given the heterogeneity in choice of outcome measures relating to measures of fertility potential, it was impossible to produce one summary measure of fertility potential. It is therefore possible that the results of the meta‐analyses do not reflect the true picture. Each of the fertility measures assessed is also only a surrogate for true fertility, and although some, such as sperm count^40^ and morphology^41^, have been correlated with fertility, others, including testicular volume, number of germ cells present on testicular biopsy and testicular histology at the time of operation, have a less certain relationship^10^ ^42^, ^43^, ^44^, ^45^. It is therefore impossible, from the studies included in this review, to draw any direct correlations between performing orchidopexy before 1 year of age and improved fertility.

A previous systematic review by Chan and colleagues^23^ summarized the evidence relating to the impact of age of orchidopexy on testicular malignancy and fertility potential. Their conclusion was that the optimal time to perform orchidopexy was between 6 and 12 months of age, as this was considered to maximize fertility potential and minimize malignancy risk, whilst also allowing for the spontaneous descent of the cryptorchid testis, which is reported to occur most commonly before the age of 6 months^46^. Their review was, however, broad in its scope, looking at general trends in the impact of age on outcome, as opposed to exploring the impact of performing orchidopexy before a specific age cut‐off. The results of the present review are in broad agreement with those of Chan *et al*.^23^, but it provides additional information relating to the safety of orchidopexy before 1 year of age. Neither review, however, has been able to comment on the safety of delivering anaesthesia for orchidopexy before the age of 1 year, with the best evidence in this area being provided instead by the GAS (General Anaesthesia compared to Spinal) trial^22^. This is a large, high‐quality RCT comparing sevoflurane anaesthesia with awake spinal anaesthesia for inguinal herniotomy in infants with gestational age at birth of more than 26 weeks and postmenstrual age at operation of less than 60 weeks. The trial is still to report on its primary outcome of IQ scores at 5 years of age, but the secondary outcome of performance on Bayley Scales of Infant and Toddler Development at 2 years of age has shown no difference between general anaesthesia and awake spinal anaesthesia. The type and duration of general anaesthesia delivered for an inguinal herniotomy is likely to be similar to that required for an orchidopexy.

The results of this systematic review and meta‐analysis suggest that there is currently insufficient evidence to advocate a wholesale move to performing all orchidopexies before 1 year of age, but there is some evidence that this may improve fertility potential. Given the equivocal state of the available evidence, in order to support the BAPU consensus statement, high‐quality RCTs must be conducted. These trials must assess harms as well as benefits, and also document the rates of spontaneous testicular descent seen in the delayed intervention group. Significant preliminary work must, however, be completed before attempting such RCTs. This will include identification of an appropriate, reliable, non‐invasive surrogate marker of fertility, and establishment of mechanisms for linking study participants to cancer registries or routinely collected data in order to identify long‐term risk of malignancy. Results from such an RCT could be combined with the final results of the GAS study to provide the robust evidence required either to support or to refute the BAPU consensus statement.

## Supporting information


**Appendix S1** Search strategy
**Table S1** Characteristics of studies excluded at full paper review
