# Supplementary material for: Systematic review and meta‐analysis comparing outcomes following orchidopexy for cryptorchidism before or after 1 year of age
Source: BJS Open. 2018 Feb 5;2(1):1–12. doi: 10.1002/bjs5.36 (PMC5952379; doi:10.1002/bjs5.36)
Supplement: Supplementary file 1 — Appendix S1 Search strategy Table S1 Characteristics of studies excluded at full paper review [file BJS5-2-1-s001.docx]

**BJS5_36**

# **Systematic review and meta-analysis comparing outcomes following orchidopexy for cryptorchidism before or after 1 year of age**

**B. S. R. Allin, E. Dumann, D. Fawkner-Corbett, C. Kwok and C. Skerritt**

**Appendix S1** Search strategy

**Embase search strategy**

1. ascending test*.mp.

2. cryptorchism/ or undescended test*.mp.

3. impalpable test*.mp.

4. cryptorchidism.mp.

5. 1 or 2 or 3 or 4

6. (testicular adj3 atrophy).mp. [mp=title, abstract, heading word, drug trade name, original title, device manufacturer, drug manufacturer, device trade name, keyword]

7. testicular atrophy.mp. or testis atrophy/

8. testis tumor/ or testis cancer/ or testicular malignancy.mp. or seminoma/

9. testicular teratoma.mp. or testis teratoma/

10. germ cell tumor/ or yolk sac tumor/ or testicular germinoma.mp.

11. testicular carcinoma.mp. or testis carcinoma/

12. testicular cancer.mp. or testis cancer/

13. Sertoli cell tumor/ or testicular adenoma.mp.

14. sex cord tumor/ or stromal cell tumour.mp. or granulosa cell tumor/ or Leydig cell tumor/

15. testicular loss.mp.

16. testis disease/ or testicular death.mp.

17. 6 or 7 or 15 or 16

18. 8 or 9 or 10 or 11 or 12 or 13 or 14

19. fertility/ or fertility.mp. or male fertility/

20. infertility/ or male infertility/ or infertility.mp.

21. infertil*.mp.

22. 19 or 20 or 21

23. neurodevelopment.mp.

24. developmental disorder/ or neuro-development.mp. or brain development/

25. child development/ or language development/ or psychosocial development/ or psychomotor development/ or development/ or physical development/ or development.mp. or "Bayley Scales of Infant Development"/ or adolescent development/ or motor development/ or mental development/

26. spermatozoon/ or spermatogenesis/ or sperm development.mp. or spermatozoon maturation/

27. 22 or 26

28. 23 or 24 or 25

29. orchidopexy.mp. or orchidopexy/

30. testicular fixation.mp.

31. 29 or 30

32. 17 or 18 or 27 or 28

33. 5 and 31 and 32

**MEDLINE Search Strategy**

1. ascending test*.mp.

2. Cryptorchidism/ or undescended test*.mp.

3. impalpable test*.mp.

4. cryptorchidism.mp.

5. 1 or 2 or 3 or 4

6. (testicular adj3 atrophy).mp. [mp=title, abstract, original title, name of substance word, subject heading word, keyword heading word, protocol supplementary concept word, rare disease supplementary concept word, unique identifier]

7. testicular atrophy.mp.

8. Seminoma/ or Testicular Diseases/ or Testicular Neoplasms/ or Dysgerminoma/ or testicular malignancy.mp. or "Neoplasms, Germ Cell and Embryonal"/ or Teratoma/

9. Teratoma/ or testicular teratoma.mp.

10. Germinoma/ or testicular germinoma.mp.

11. Carcinoma/ or testicular carcinoma.mp.

12. testicular cancer.mp.

13. Adenoma/ or testicular adenoma.mp. or Leydig Cell Tumor/

14. Granulosa Cell Tumor/ or stromal cell tumour.mp.

15. 8 or 9 or 10 or 11 or 12 or 13 or 14

16. testicular loss.mp. or Testicular Diseases/

17. Testis/ or testicular death.mp.

18. 6 or 7 or 16 or 17

19. Fertility Preservation/ or Fertility/ or fertility.mp.

20. infertility.mp. or Infertility/ or Infertility, Male/

21. infertil*.mp. or Spermatozoa/

22. Spermatocytes/ or sperm development.mp. or Spermatogenesis/

23. 19 or 20 or 21 or 22

24. Child Development/ or Developmental Disabilities/ or neurodevelopment.mp. or Child Development Disorders, Pervasive/

25. neuro-development.mp. or Developmental Disabilities/

26. Musculoskeletal Development/ or development.mp. or Adolescent Development/ or Language Development/ or "Growth and Development"/ or Language Development Disorders/ or Human Development/ or Personality Development/ or Child Development/

27. 24 or 25 or 26

28. orchidopexy.mp. or Orchiopexy/

29. testicular fixation.mp.

30. 28 or 29

31. 15 or 18 or 23 or 27

32. 5 and 30 and 31

**Table S1** Characteristics of studies excluded at full paper review

| **Study** | **Setting and Methodology** | **Reasons for exclusion** |
| --- | --- | --- |
| Al Saywid 2011 | Retrospective cohort study/ case series, Australia | No active comparison between infants undergoing orchidopexy prior to one year of age, and those undergoing orchidopexy after one year of age. |
| Beltran-Brown 1988 | Retrospective cohort study/ case series, Mexico | No active comparison between infants undergoing orchidopexy prior to one year of age, and those undergoing orchidopexy after one year of age. |
| Bianchi 1995 | Expert review, United Kingdom | Limitations of study type |
| Bilius 2015 | Retrospective cohort study/ case series, Lithuania | No active comparison between infants undergoing orchidopexy prior to one year of age, and those undergoing orchidopexy after one year of age (comparison between infants operated on prior to or after 1.5 years of age). |
| Chan 2014 | Systematic review | No active comparison between infants undergoing orchidopexy prior to one year of age, and those undergoing orchidopexy after one year of age. |
| Coughlin 1999 | Retrospective cohort study/ case series, United States | No active comparison between infants undergoing orchidopexy prior to one year of age, and those undergoing orchidopexy after one year of age. |
| Docimo 2000 | Expert review | Limitations of study type |
| Ein 2014 | Retrospective cohort study/ case series, Canada | No active comparison between infants undergoing orchidopexy prior to one year of age, and those undergoing orchidopexy after one year of age. Limitations of methodology - syndromic cases and operation for torsion not excluded. |
| Engeler 2000 | Retrospective cohort study/ case series, Switzerland | No active comparison between infants undergoing orchidopexy prior to one year of age, and those undergoing orchidopexy after one year of age (comparison between infants operated on prior to or after 2 years of age). |
| Gapany 2008 | Systematic review | No active comparison between infants undergoing orchidopexy prior to one year of age, and those undergoing orchidopexy after one year of age. |
| Gracia 2000 | Retrospective cohort study/ case series, Spain | No active comparison between infants undergoing orchidopexy prior to one year of age, and those undergoing orchidopexy after one year of age. |
| Hack 2007 | Prospective case series, Denmark | No active comparison between infants undergoing orchidopexy prior to one year of age, and those undergoing orchidopexy after one year of age. |
| Hadziselimovic 2001_1 | Retrospective cohort study/ case series, Switzerland | No active comparison between infants undergoing orchidopexy prior to one year of age, and those undergoing orchidopexy after one year of age (comparison between infants operated on prior to or after 6 months of age). |
| Hadziselimovic 2001_2 | Retrospective cohort study/ case series, Switzerland | No active comparison between infants undergoing orchidopexy prior to one year of age, and those undergoing orchidopexy after one year of age (comparison between infants operated on prior to or after 6 months of age). |
| Herrington 2003 | Case-control study, United States | No active comparison between infants undergoing orchidopexy prior to one year of age, and those undergoing orchidopexy after one year of age. |
| Hrivatakis 2014 | Retrospective cohort study/ case series, Germany | No active comparison between infants undergoing orchidopexy prior to one year of age, and those undergoing orchidopexy after one year of age. |
| Kapelari 2007 | Expert review | Limitations of study type |
| Kelsberg 2006 | Expert review | Limitations of study type |
| Kohva 2015 | Retrospective cohort study/ case series, Finland | No active comparison between infants undergoing orchidopexy prior to one year of age, and those undergoing orchidopexy after one year of age. |
| Lee 1996 | Retrospective cohort study/ case series, United States | No active comparison between infants undergoing orchidopexy prior to one year of age, and those undergoing orchidopexy after one year of age. |
| Lee 1997 | Retrospective cohort study/ case series, United States | No active comparison between infants undergoing orchidopexy prior to one year of age, and those undergoing orchidopexy after one year of age. |
| Lee 2002 | Retrospective cohort study/ case series, United States | No active comparison between infants undergoing orchidopexy prior to one year of age, and those undergoing orchidopexy after one year of age. |
| Lee 2005 | Retrospective cohort study/ case series, United States | No active comparison between infants undergoing orchidopexy prior to one year of age, and those undergoing orchidopexy after one year of age. |
| Lim 2003 | Retrospective cohort study/ case series, Ireland (ROI) | No active comparison between infants undergoing orchidopexy prior to one year of age, and those undergoing orchidopexy after one year of age. |
| Miller 2001 | Retrospective cohort study/ case series, United States | No active comparison between infants undergoing orchidopexy prior to one year of age, and those undergoing orchidopexy after one year of age. |
| Nagar 1997 | Retrospective cohort study/ case series, Israel | No active comparison between infants undergoing orchidopexy prior to one year of age, and those undergoing orchidopexy after one year of age (comparison between infants operated on prior to or after 1.5 years of age). |
| Nah 2014 | Retrospective cohort study/ case series, Singapore | No active comparison between infants undergoing orchidopexy prior to one year of age, and those undergoing orchidopexy after one year of age. |
| Shoukry 2012 | Retrospective cohort study/ case series, United Kingdom | No active comparison between infants undergoing orchidopexy prior to one year of age, and those undergoing orchidopexy after one year of age. |
| Sumiya 1991 | Retrospective cohort study/ case series, Japan | No active comparison between infants undergoing orchidopexy prior to one year of age, and those undergoing orchidopexy after one year of age. |
| Thorup 2007 | Expert review | Limitations of study type |
| Tasian 2009 | Retrospective cohort study/ case series, United States | No active comparison between infants undergoing orchidopexy prior to one year of age, and those undergoing orchidopexy after one year of age. |
| Thorup 2011 | Retrospective cohort study/ case series, Denmark | No active comparison between infants undergoing orchidopexy prior to one year of age, and those undergoing orchidopexy after one year of age. |
| Tzvetkova 1996 | Retrospective cohort study/ case series, Bulgaria | No active comparison between infants undergoing orchidopexy prior to one year of age, and those undergoing orchidopexy after one year of age (no patients operated on before age 9 years). |
| van der Plas 2015 | Retrospective cohort study/ case series, The Netherlands | No active comparison between infants undergoing orchidopexy prior to one year of age, and those undergoing orchidopexy after one year of age (comparison between children operated on prior to age 8 years of after age 12). |
